# Unusual presentation of anomalous origin of the right pulmonary artery from the ascending aorta: case report

**DOI:** 10.1186/s43044-025-00614-6

**Published:** 2025-02-04

**Authors:** Mohamed Elhudairy, Naif Alkhushi, Osman Al-Radi, Khadijah Maghrabi, Gaser Abdelmohsen

**Affiliations:** 1 Pediatric Cardiology Division, Department of Pediatrics, King Abdul-Aziz University, P.O. Box: 80215, 21589 Jeddah, Saudi Arabia; 2 Cardiac Surgery Division, Department of Surgery, King Abdulaziz University, P.O. Box: 80215, 21589 Jeddah, Saudi Arabia; 3 Pediatric Cardiology Division, Department of Pediatrics, Kasr Al Ainy School of Medicine, Cairo University, Cairo, 11562 Egypt

**Keywords:** AORPA, Anomalous pulmonary artery, PDA, Prostaglandin E1 bolus, Congenital heart defects, Pulmonary hypertension

## Abstract

**Background:**

Anomalous origin of the right pulmonary artery (AORPA) from the ascending aorta is a rare congenital anomaly, representing approximately 0.12% of all congenital heart defects. Early diagnosis and timely intervention are essential to prevent severe complications such as heart failure and pulmonary vascular disease.

**Case presentation:**

We report a case of a full term neonate presented with respiratory distress and cyanosis. Echocardiography revealed an anomalous right pulmonary artery (RPA) origin from the ascending aorta, a large patent ductus arteriosus (PDA) with right-to-left shunt, and moderate tricuspid regurgitation. Despite initial management with prostaglandin E1 (PGE1) infusion, discontinuation of the drug led to clinical deterioration characterized by severe metabolic acidosis and low cardiac output syndrome. Resuming PGE1 infusion stabilized the patient’s hemodynamics and improved systemic blood flow, allowing for successful surgical repair.

**Conclusion:**

In cases of AORPA associated with aortic arch flow reversal, pulmonary hypertension, and inadequate interatrial communication, maintaining PDA patency with PGE1 infusion until surgical repair is critical for survival. The right-to-left flow across the PDA counteracts the steal from the aorta and decompresses the right ventricle, preventing right ventricular failure and maintaining systemic blood flow.

**Graphical abstract:**

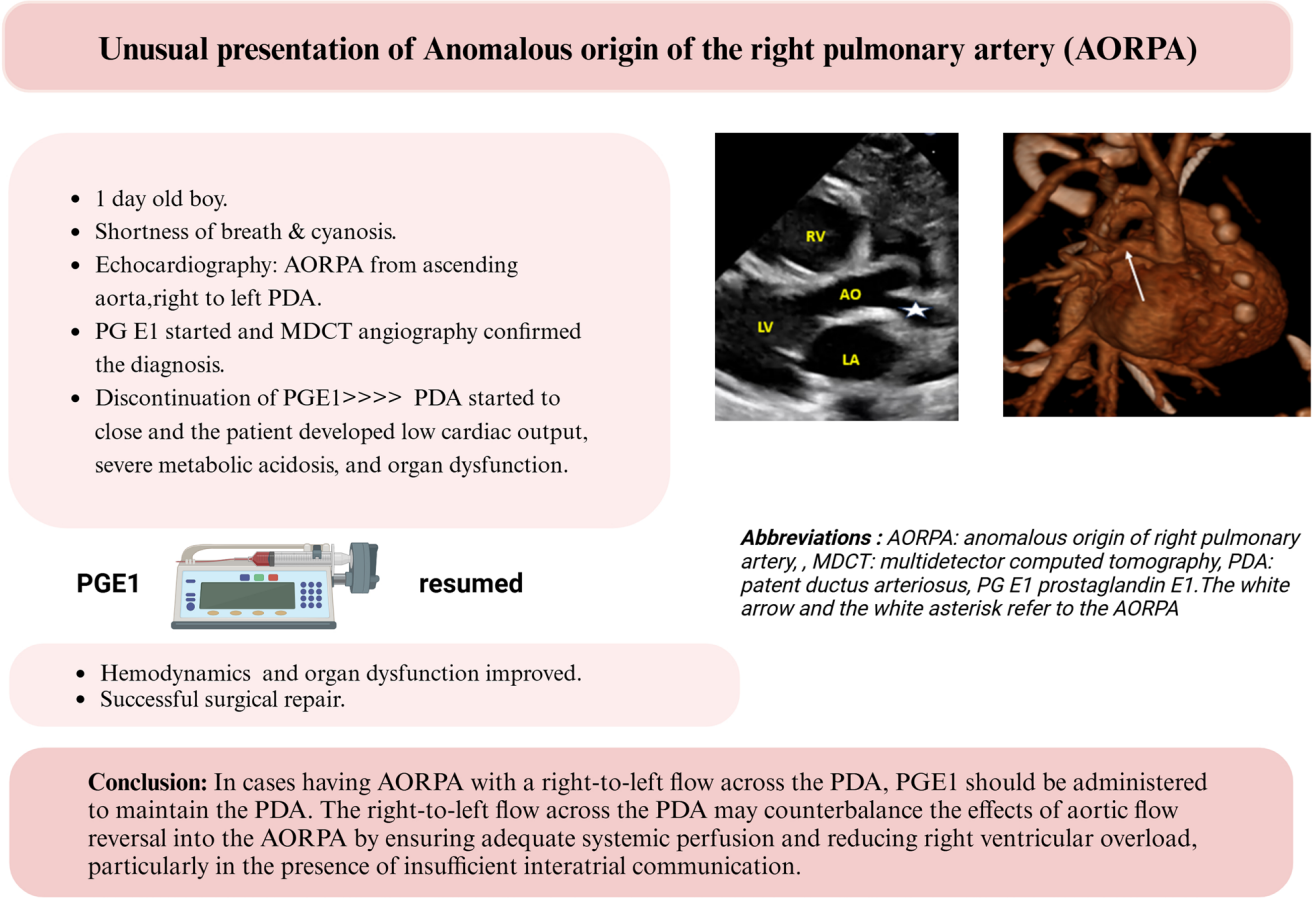

**Supplementary Information:**

The online version contains supplementary material available at 10.1186/s43044-025-00614-6.

## Background

The lungs can get an aberrant blood supply from several sources, including major aortopulmonary collaterals (MAPCAS), patent ductus arteriosus (PDA), common arterial trunk, coronary arteries, or through the anomalous origin of the pulmonary arteries from the aorta [1]. Anomalous origin of the right pulmonary artery (AORPA) comprises approximately 0.12% of all congenital cardiac defects and has the potential to be fatal if not diagnosed early. Although the AORPA typically originates from the ascending aorta, it may also arise from the descending aorta. AORPA can be associated with a right-sided aortic arch, anomalous subclavian artery, tetralogy of fallot (TOF), heterotaxy, and patent ductus arteriosus (PDA) [2, 3]. PDA may be of utmost importance in such circumstances, mainly if there is pulmonary hypertension with a significant diastolic steal from the aorta to the anomalous right pulmonary artery (RPA) with inadequate interatrial communication. Early diagnosis of such patients is crucial since the one-year survival rate is less than 30% if not recognized and treated early. Furthermore, most untreated cases will be complicated by irreversible pulmonary vascular disease, heart failure, and death. AORPA may be challenging to diagnose and readily overlooked; alternative imaging modalities besides transthoracic echocardiogram (TTE) may be required for an accurate diagnosis, and early surgical correction is recommended [4, 5].

## Case presentation

**Fig. 1:**
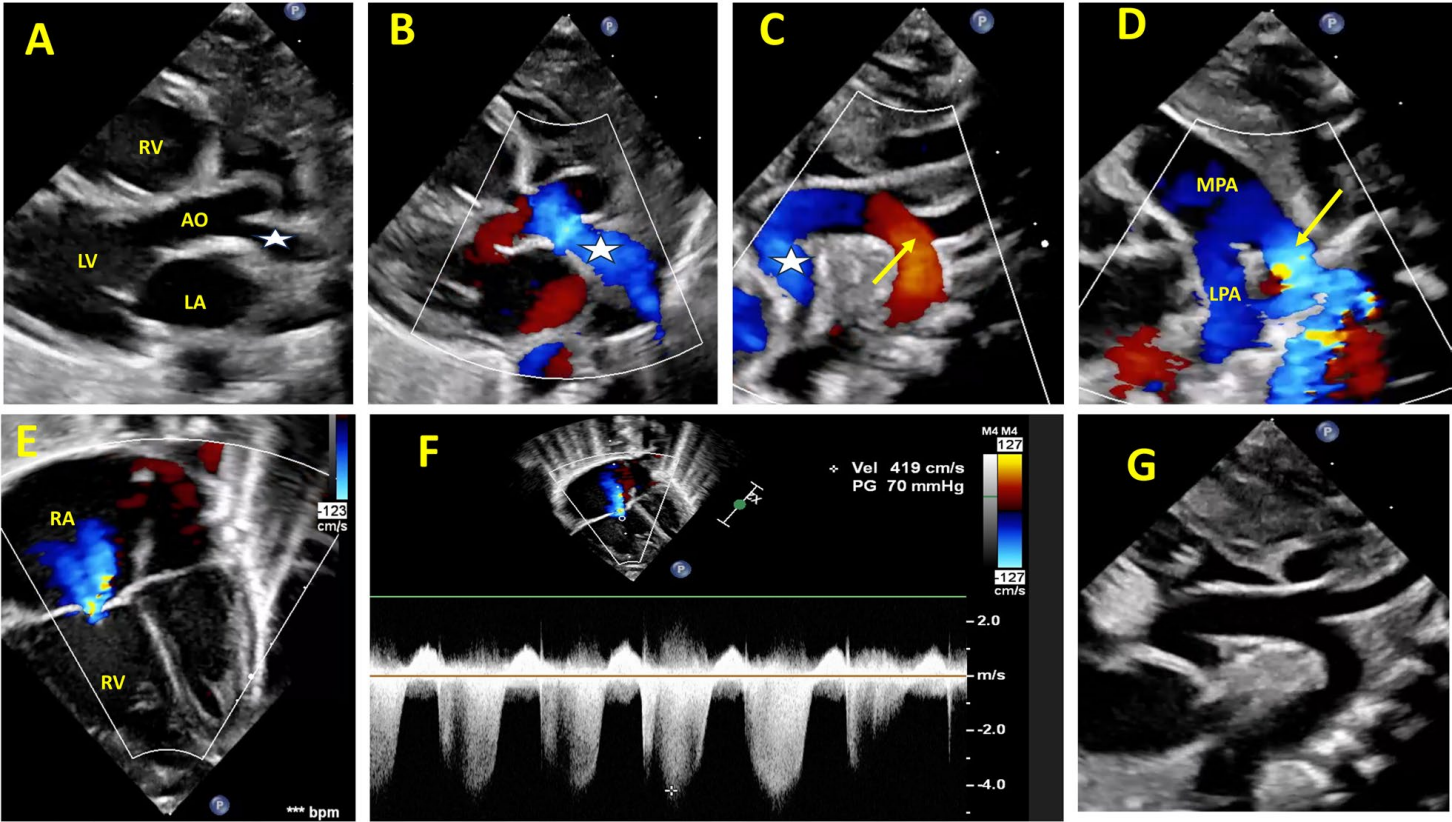
**A** 2D image of parasternal long axis view showing the RPA arising from the ascending aorta (white asterisk). **B** Color Doppler of parasternal long axis view showing the RPA (white asterisk). **C:** Suprasternal long axis view showing RPA (white asterisk) and the retrograde flow in the aortic arch (yellow arrow). **D** The parasternal short-axis view shows the right-to-left PDA flow (yellow arrow). **E** An apical four-chamber view shows moderate tricuspid regurgitation (TR) with dilated RA and RV **F** Continuous wave Doppler at TR showing a TR gradient of 70 mmHg (indicating severe pulmonary hypertension). **G** Suprasternal long axis view showing the aortic arch and the proximal descending aorta with no coarctation. Ao: aorta: LA: left atrium, LPA: left pulmonary artery, LV: left ventricle, MPA: mean pulmonary artery, PDA: patent ductus arteriosus, RA: right atrium, RPA: right pulmonary artery, RV: right ventricle, TR: tricuspid regurgitation

**Fig. 2:**
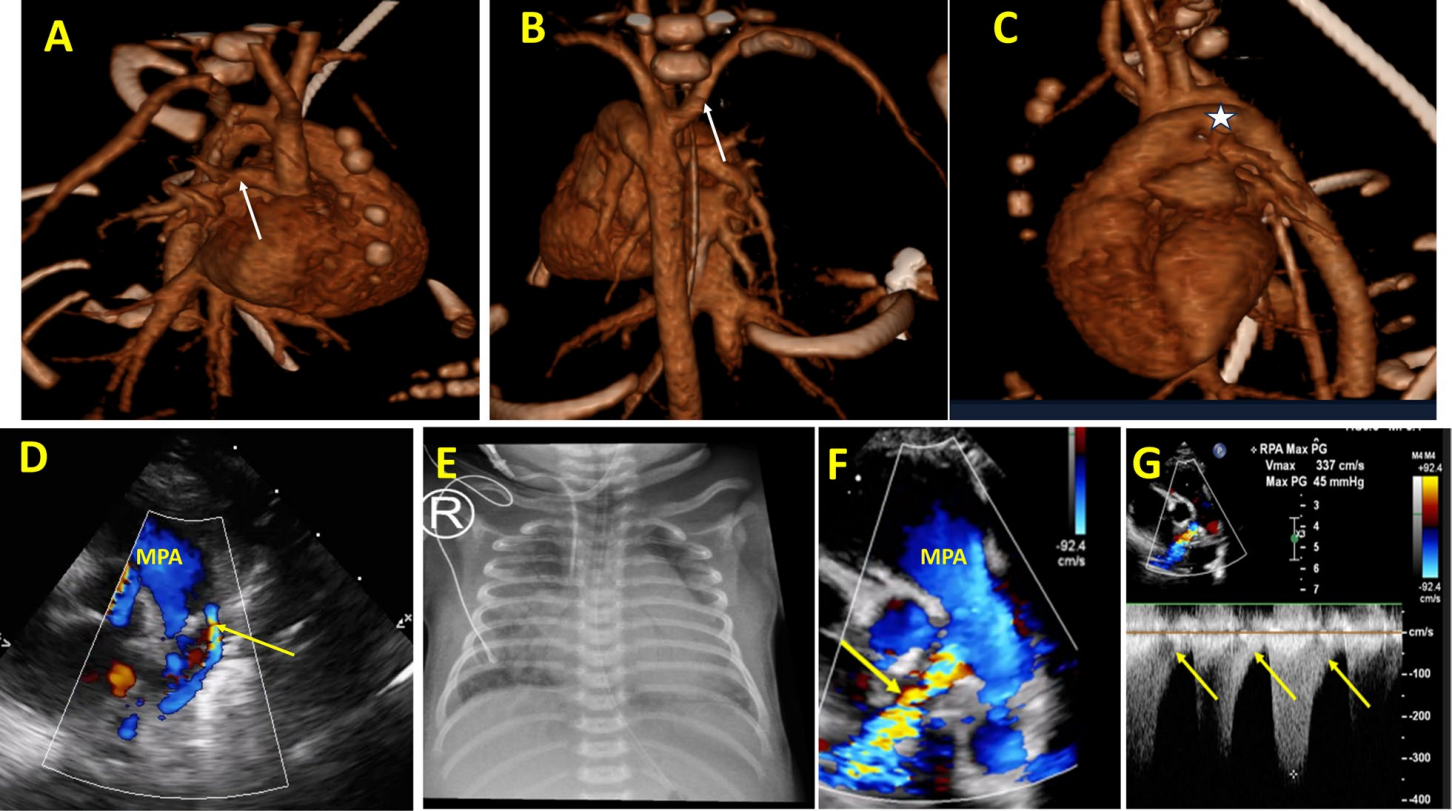
**A** MDCT angiography with 3D reconstruction and right lateral projection showing the anomalous RPA from the ascending aorta (white arrow). **B** MDCT angiography with 3D reconstruction and posterior projection showing the aberrant right subclavian artery (white arrow) with no coarctation of the aorta. **C** MDCT angiography with 3D reconstruction and left lateral projection showing the large PDA (white arrow) connected to the descending aorta. **D** The parasternal short-axis view shows constriction of the PDA with right-to-left shunting after the discontinuation of prostaglandins (yellow arrow). **E** Preoperative chest x-ray showing plethoric right lung. **F** The parasternal short-axis view shows postoperative RPA stenosis (yellow arrow). **G** continuous wave Doppler showing postoperative RPA stenosis with diastolic runoff (yellow arrows). MDCT: multidetector computed tomography, MPA: mean pulmonary artery, PDA: patent ductus arteriosus, RPA: right pulmonary artery

A 38-week gestational age baby boy, a product of a non-consanguineous marriage, arrived at our hospital’s emergency department (ED) on his first day of life, exhibiting signs of tachypnea and cyanosis. Upon arrival, a nasal cannula was connected to the patient with an oxygen flow 2L/m. Examination revealed no apparent dysmorphic features. The patient had respiratory distress (respiratory rate 80/min) with intercostal and subcostal recessions and tachycardia (heart rate 170–190/min). Peripheral pulses were intact. The cardiovascular examination revealed differential cyanosis (oxygen saturation in the left hand was 90%, and in the right hand and foot was 85% and 75%, respectively), intact peripheral pulsations, sternal heave with normal first heart sound (S1), accentuated second heart sound (S2), and a continuous machinery murmur heard over the infraclavicular area. Additionally, the liver was palpable 2 cm below the right costal margin. No significant blood pressure gradient was observed between the four limbs. Prostaglandin E1(PGE1) infusion started (0.05 mcg/kg/min) and was connected to continuous positive airway pressure (CPAP). Echocardiography revealed patent foramen ovale (PFO) with bidirectional shunting, moderate tricuspid regurgitation (TR) with estimated right ventricle systolic pressure (RVSP) of 70 mmHg, dilated right ventricle (RV), right atrium, large PDA with a right-to-left shunt, and diastolic reversal flow in the aortic arch, with no coarctation of the aorta. The RPA anomalously arose from the ascending aorta (Fig. 1A–G, video 1). Due to the significant flow reversal in the aortic arch, a bedside cranial ultrasound was performed, which excluded brain arteriovenous malformation. Multidetector computed tomography (MDCT) revealed an anomalous origin of RPA (5.5 mm) from the proximal ascending aorta, large PDA (7.5 mm) with a focal narrowing at the pulmonary arterial connection to a diameter of 5 × 3.5 mm, and left-sided aortic arch with an aberrant right subclavian artery with no focal aortic coarctation (Fig. 2A–C). After MDCT, we thought the PGE1 was unnecessary after excluding aortic coarctation or aortic arch interruption. We thought that the PGE1 may increase the steal from the aorta to the AORPA by decreasing the pulmonary vascular resistance, so prostaglandin E1 (PGE1) was discontinued. Following the cessation of PGE1, the patient’s condition progressively worsened over the following 8 h. The patient had more tachypnea, low cardiac output manifested by hypotension (BP 45/30 mmHg), oliguria, and hepatomegaly with liver 4 cm below the right costal margin, and arterial blood gas done at that time revealed severe metabolic acidosis with PH 6.9, HCO3 5 mmol/l, lactate 18 mmol/l, and BE −20. The patient was intubated, and an echocardiogram was performed immediately after deterioration, which revealed a closing ductus arteriosus along with a significantly reduced blood flow in the descending aorta and a dilated right ventricle (RV) with poor systolic function (Fig. 2D, video 2), and small PFO with right-to-left shunt. After this deterioration, a prostaglandin bolus was administered (0.2 mcg/kg), followed by continuous intravenous infusion, which was resumed at 0.2 mcg/kg/min. Additionally, epinephrine infusion was initiated at 0.05 mcg/kg/min. Ten minutes later, echocardiography revealed a large PDA with good flow and pulsatility in the descending aorta. A few hours later, the patient’s hemodynamics markedly improved, the patient’s blood pressure gradually increased to 65–70/40 mmHg (mean 50 mmHg), and the preductal oxygen saturation (in the left hand) was 85%, and then increased to 95%. Additionally, the patient’s urine output increased, and metabolic acidosis and hyperlactatemia gradually improved until they returned to normal. After this notable improvement, PGE1 was gradually tapered to 0.05 mcg/kg/min. Following this incident, the patient experienced renal and hepatic injury, and their liver and kidney function tests (LFTs and KFTs) were impaired. It took a few days for LFTs and KFTs to return to baseline after stabilizing the patient’s condition. The patient then underwent surgical repair in which deep hypothermic circulatory arrest was achieved with the body temperature lowered to 18 degrees Celsius. The PDA was divided. The arch appeared unremarkable from its exterior. The ascending aorta was surgically divided, and the right pulmonary artery (RPA) was separated from it. A surgical incision was made on the right side of the MPA. The RPA was anastomosed to MPA. A portion of the equine pericardium was used to augment the proximal RPA. CPB was gradually discontinued following successful hemostasis. Two intercostal drainage tubes were placed. The chest was covered for delayed closure. The patient was transferred to the pediatric cardiac intensive care unit (PCICU) with an open sternum. The patient’s hemodynamic status remained stable, and the sternum was closed two days later. The patient underwent extubation and transitioned smoothly to noninvasive ventilation (NIV), followed by a successful transition to room air. The initial postoperative echocardiogram revealed a peak systolic pressure gradient of 24 mmHg on the RPA with normal cardiac function. The patient was discharged home and returned in good condition for a follow-up appointment at the outpatient department after two months. Echocardiography was done in the clinic that revealed severe RPA stenosis, and the patient was booked for cardiac catheterization for balloon angioplasty (Fig. 2F–G, video 3).

## Discussion

The earliest description of AORPA was provided by Fraentzel in 1868. However, the process of AORPA embryogenesis is still not fully understood. Some studies have shown a relationship between its occurrence and a defect in the migration of neural crest cells during septation of the common arterial trunk into the aorta and pulmonary artery, as seen in cases with 22q 11 deletions [3, 6]. In cases of AORPA, immediately after birth, with the physiological decrease in the pulmonary vascular resistance (PVR), a significant steal of blood flow from the aorta to the AORPA can happen. In our case, the AORPA originated from the ascending aorta and resulted in a substantial steal of blood flow from the descending aorta to the AORPA; this steal may be increased after initiating PGE1 as prostaglandin can decrease the PVR. This steal led to a notable and significant retrograde flow in the aortic arch and descending aorta seen during echocardiographic examinations (Fig. 1, video 1). These hemodynamic changes were evident in the plethoric right lung (Fig. 2E).

The patient has an aberrant right subclavian artery originating from the proximal descending aorta and a right-to-left shunt via the patent ductus arteriosus (PDA). As a result, the oxygen saturation in the right hand and lower limbs was lower compared to the left hand. In this situation, the saturation levels in the right hand and lower extremities represent postductal oxygen saturation, whereas the saturation level in the left hand represents preductal saturation.

The patient’s conditions worsened after stopping PGE1, resulting in low cardiac output syndrome and reduced flow in the descending aorta, as observed through echocardiography. There are two possibilities to explain this deterioration: the first possibility is the presence of severe coarctation of the aorta (COA) or interrupted aortic arch (IAA), leading to systemic circulation that relies on the right-to-left ductal flow. The absence of COA/IAA by the MDCT ruled out this possibility. The second possibility for the deterioration observed after discontinuation of prostaglandin E1 could be attributed to the existence of a patent ductus arteriosus (PDA) that allows blood to flow primarily from the left pulmonary artery (LPA) to the descending aorta (DAO). This flow may counteract and balance the significant reversal/ steal of blood flow from the aortic arch and DAO into the anomalously right pulmonary artery (RPA), thereby maintaining systemic circulation.

Moreover, the PDA may also decompress the RV in the presence of small PFO/ASD. The right-to-left flow across the ASD can also maintain the systemic flow if the ASD/PFO is of adequate size, a condition not present in our case. The absence of COA/IAA in MDCT and intraoperatively makes the second possibility more logical.

Therefore, the patient’s deterioration can be attributed to the closure of the ductus arteriosus in the presence of severe pulmonary hypertension (in the left lung), which caused increased pressure overload to the right ventricle (RV) and closure of the main vent of the RV (as PFO was small). The acute increase in RV afterload with small interatrial communication to vent the right side was clinically confirmed by a significant increase in liver span and by TTE, which revealed a worsening TR and severe RV dilatation and dysfunction. Moreover, the diminished blood flow in the descending aorta leads to an insufficient supply of blood to different organs, such as the kidneys, liver, and gut, resulting in the malfunctioning of these organs. However, their function improved following the resumption of PGE1 with the reopening of the PDA and the restoration of blood flow in the descending aorta.

MDCT angiography and cardiac magnetic resonance imaging are essential for diagnosing AORPA because they provide comprehensive anatomical details beyond the limitations of echocardiography, particularly in situations with inadequate acoustic windows [5]. Early surgical treatment of AORPA is of paramount importance as untreated cases have a higher risk for mortality and morbidity due to the development of heart failure and pulmonary vascular obstructive disease. Postoperative long-term follow-up for these cases is vital due to the risk of pulmonary branch stenosis at the suture lines of AORPA to the main pulmonary artery [2–4, 7], as happened with this patient and illustrated in Fig. 2F–G and video 3.

## Conclusion

In cases where AORPA is accompanied by aortic arch flow reversal and pulmonary hypertension with right-to-left ductal flow, the use of PGE1 infusion to maintain ductal patency until surgical intervention could be critical for survival. The right-to-left flow across the PDA may counterbalance the effects of aortic flow reversal into the AORPA by ensuring adequate systemic perfusion and reducing right ventricular overload, particularly in the presence of insufficient interatrial communication.

## Supplementary Information

Additional file 1: Video 1. Echocardiography was done while the patient was on prostaglandin infusion, showing anomalous RPA from ascending aorta with retrograde flow in the aortic arch and descending aorta, right to left PDA flow with no coarctation of the aorta, moderate TR with dilated RV and RA. RPA: right pulmonary artery, PDA: patent ductus arteriosus, RA: right atrium, RV: right ventricle, TR: tricuspid regurgitation.

Additional file 2: Video 2. Echocardiography done after discontinuation of prostaglandins showing constriction of the PDA with right to left PDA flow, moderate TR with RA and RV dilatation, RV compressing the LV, ASD with bidirectional shunting. ASD: atrial septal defect, LV: left ventricle, PDA: patent ductus arteriosus, RA: right atrium, RV: right ventricle, TR: tricuspid regurgitation.

Additional file 3: Video 3. Echocardiography was done postoperatively in the clinic, showing significant RPA stenosis with diastolic flow also seen across the RPA. RPA: right pulmonary artery. 

## Data Availability

All data and materials will be uploaded as per the needs of the editor/reviewer or the readers as per their request.

## Abbreviations

AORPA: Anomalous origin of the right pulmonary artery
ASD: Atrial septal defect
COA: Coarctation of the aorta
CPAP: Continuous positive airway pressure
CPB: Cardiopulmonary bypass
IAA: Interrupted aortic arch
KFTs: Kidney function tests
LFTs: Liver function tests
LPA: Left pulmonary artery
LV: Left ventricle
MDCT: Multidetector computed tomography
MPA: Main pulmonary artery
NIV: Noninvasive ventilation
PDA: Patent ductus arteriosus
PGE1: Prostaglandin E1
PCICU: Pediatric cardiac intensive care unit
PFO: Patent foramen ovale
RA: Right atrium
RPA: Right pulmonary artery
RV: Right ventricle
TR: Tricuspid regurgitation
TTE: Transthoracic echocardiogram
